# Foveal avascular zone and macular vessel density after correction for magnification error in unilateral amblyopia using optical coherence tomography angiography

**DOI:** 10.1186/s12886-019-1177-z

**Published:** 2019-08-05

**Authors:** Syunsuke Araki, Atsushi Miki, Katsutoshi Goto, Tsutomu Yamashita, Tsuyoshi Yoneda, Kazuko Haruishi, Yoshiaki Ieki, Junichi Kiryu, Goro Maehara, Kiyoshi Yaoeda

**Affiliations:** 1 Department of Ophthalmology, Kawasaki Medical School, 577 Matsushima, Kurashiki, Okayama, 701-0192 Japan; 2 Doctoral Program in Sensory Science, Graduate School of Health Science and Technology, Kawasaki University of Medical Welfare, 288 Matsushima, Kurashiki, Okayama, 701-0193 Japan; 3 Department of Sensory Science, Faculty of Health Science and Technology, Kawasaki University of Medical Welfare, 288 Matsushima, Kurashiki, Okayama, 701-0193 Japan; 4 Department of Human Sciences, Kanagawa University, 3-27-1 Rokkakubashi, Yokohama, Kanagawa 221-8686 Japan; 5 Yaoeda Eye Clinic, 2-1649-1 Naga-Chou, Nagaoka, Niigata, 940-0053 Japan

**Keywords:** Amblyopia, Retina, Optical coherence tomography angiography, Foveal avascular zone, Macular vessel density

## Abstract

**Background:**

To investigate the area of foveal avascular zone (FAZ) and macular vessel density (VD) after correction for magnification error in unilateral amblyopia using optical coherence tomography angiography (OCTA).

**Methods:**

Participants comprised 15 patients with unilateral amblyopia due to anisometropia with or without strabismus (mean age, 9.8 ± 3.4 years; range, 6–17 years). OCTA images were obtained by using spectral-domain OCT with angiography software. The OCTA scanning protocol used was 3 × 3-mm volume scan centered on the fovea. OCTA images were corrected for magnification errors using individual axial length (AL), and an adjusted 2.3 × 2.3-mm square was derived as a region of interest. The FAZ area and VD in both superficial capillary plexus (SCP) and deep capillary plexus (DCP) layers, foveal minimum thickness (FMT) were assessed using built-in OCTA software and ImageJ software (NIH, Bethesda, MD).

**Results:**

LogMAR in the amblyopic eyes was significantly poorer than that of the fellow eye (*p* < 0.001). AL was significantly shorter in the amblyopic eye than in the fellow eye (*p* < 0.001). FAZ area of SCP in amblyopic eyes was significantly smaller than that of fellow eyes (*p* < 0.001). No significant differences were seen in FAZ area of DCP, VD of SCP, VD of DCP, and FMT between amblyopic and fellow eyes (*p* = 0.07, 0.43, 0.55, and 0.25, respectively).

**Conclusions:**

Our present study after magnification error correction found smaller FAZ area of SCP in the amblyopic eye compared with the fellow eyes, but there was no significant difference in the macular VD between the amblyopic and fellow eyes.

## Background

Amblyopia is a common developmental disorder involving dysfunctional processing of visual information, primarily characterized by reduced visual acuity, and resulting from either disuse due to the absence of a clear image on the retina, or misuse due to abnormal binocular interactions [1]. On the pathology of amblyopia, although conventional clinical ocular examination is most often entirely normal in amblyopia, microscopic morphological and functional abnormalities have been found in the visual cortex and lateral geniculate nucleus [2–4]. On the other hand, although abnormalities of the retina in amblyopia, such as dysfunction of retinal ganglion cells, have been indicated [5, 6], scientific evidence has been lacking [7].

In recent years, detailed studies on the retinal morphology of amblyopic eyes of in vivo imaging have been conducted using optical coherence tomography (OCT), and the retinal abnormalities in amblyopia are being discussed again. Our previous study [8] reported no significant difference in foveal and macular inner retinal thicknesses of patients with unilateral amblyopia and that retinal thicknesses were unrelated to the degree of amblyopia. In contrast, Li et al. showed that the foveolar thickness in amblyopic eyes is thicker than that of normal control eyes [9]. In addition, a relationship between visual function and retinal morphology has been shown by comparisons between before and after amblyopia treatment [10, 11]. Consensus on abnormalities of the retina in amblyopia has yet to be obtained.

More recently, OCT angiography (OCTA) has been developed as a noninvasive imaging technique that allows mapping of the retinal microvasculature [12]. As the OCTA does not use dye injection and can be performed in a short period of time, there are virtually no side effects and it is straightforward to perform OCTA even in children. OCTA allows stratified evaluation of the retinal vasculature, which is difficult to evaluate with conventional fluorescein angiography. In addition, as the boundary of the blood vessel can be clearly depicted, foveal avascular zone (FAZ) area and vessel density (VD) can be quantitatively analyzed. Characteristic morphological changes in the FAZ or VD in retinal vascular diseases such as diabetic retinopathy (DR) and retinal venous occlusion (RVO) have been reported [13, 14]. However, few reports appear to have examined the retinal vasculature using OCTA in amblyopia [15–18] and none of those studies paid special attention to differences in axial length (AL) or refraction of amblyopic eyes, which are often hyperopic and small, in the data analysis. We therefore evaluated FAZ area and macular VD after magnification error correction using OCTA in unilateral amblyopia to investigate the retinal vasculature of amblyopic eyes.

## Methods

This study adhered to the tenets of the Declaration of Helsinki and was approved by the institutional review board committee at Kawasaki Medical School (registration number: 3473). This study was designed as a cross-sectional study and conducted from August 2017 to September 2017 in the Department of Ophthalmology at Kawasaki Medical School Hospital. This study included minors under the age of 16 in the patient sample. Oral informed consent for the examinations was obtained from each patient or one of the parents of each patient.

### Subjects

All enrolled patients were diagnosed with unilateral amblyopia due to anisometropia or anisometropia combined with strabismus (mixed-type) and underwent OCTA examination. Ophthalmologic examinations performed in all patients included best-corrected visual acuity (BCVA), intraocular pressure, cycloplegic refraction (RKT-7700; NIDEK, Gamagori, Japan), AL (IOL Master^®^; Carl Zeiss Meditec AG, Jena, Germany), cover test, extraocular movements, slit-lamp, and fundoscopy. Anisometropic (or mixed) amblyopia was defined as a condition in which decimal BCVAs were less than 0.8 in the amblyopic eye due to anisometropia (including anisometropia combined with strabismus), defined as an interocular difference in refraction (spherical equivalent) of more than 2.0 diopters (D), and more than 1.0 in the fellow eye at the first visit. Exclusion criteria were as follows: history of ocular diseases, history of intraocular surgery, and presence of systemic diseases that that may have had an influence on the eye. The presence or absence of history of amblyopia treatment at OCTA examination was not taken into consideration in the data analysis.

### OCT scan protocol and image analysis

**Fig. 1:**
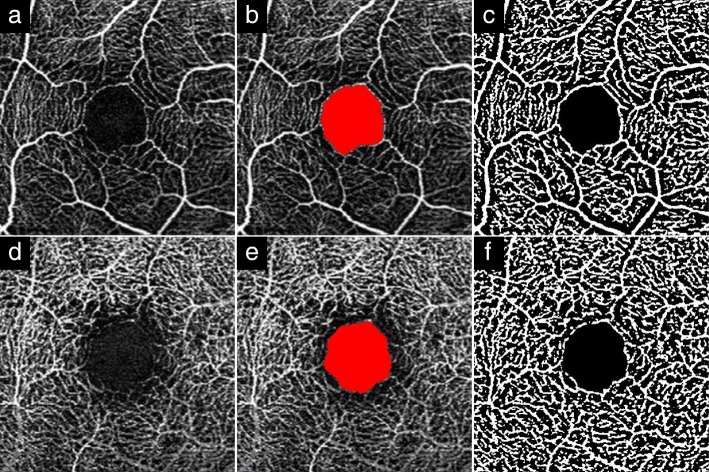
Analysis of optical coherence tomography angiography image. Typical optical coherence tomography angiography images from a patient. **a**; Superficial capillary plexus (SCP) image. **b**; Foveal avascular zone (FAZ) area in SCP (region shown in red). **c**; Binarized OCTA image using ImageJ in SCP. **d**; Deep capillary plexus (DCP) image. **e**; FAZ area in DCP (region shown in red). **f**; Binarized OCTA image using ImageJ in DCP. Vascular densities in SCP and DCP were calculated from binary images in **c** and **f**

OCTA images were obtained using spectral-domain (SD)-OCT with software (RS-3000 Advance with AngioScan2 software; NIDEK, Gamagori, Japan) (Fig. 1a, d). Specifications for this SD-OCT were: light source wavelength, 880 nm; resolution, 20 × 7 μm; and scan speed, 53,000 A-scans/s. The OCTA scanning protocol used was a 3 × 3-mm volume scan centered on the fovea using the tracing HD function. Scan density was 256 clusters of four repeated B-scans. Data from SD-OCT were corrected for the AL-related magnification using built-in software to take into account the FAZ area and VD measurement error due to image magnification [19]. All SD-OCT examinations were performed by a single experienced technician (S.A.) under non-mydriasis between 9:00 AM and 12:00 PM.

FAZ area (mm^2^) was defined as the avascular area in the center of the fovea, and the border of the FAZ was manually measured in each patient by two examiners (S.A., K.G.) using software equipped with the device. FAZ area values were scaled by the individual magnification correction factor to obtain the corrected FAZ area with reference to the report by Sampson et al. [19]. The average of magnification-corrected FAZ areas from two measurements was used for analysis. We separately evaluated FAZ areas in both the superficial capillary plexus (SCP) and deep capillary plexus (DCP) layers (Fig. 1b, e). Based on the default settings, the SCP layer was defined from the top of the inner limiting membrane to 8 μm below the bottom of the inner plexiform layer. The DCP layer was defined from 13 to 88 μm below the inner plexiform layer.

Macular VD (%) was calculated using ImageJ software (version 1.51j8; National Institute of Health, Bethesda, MD) according to previous studies [20, 21]. A 2.3 × 2.3-mm square was derived as a region of interest from an OCTA image scaled by the magnification correction factor using open source image-processing software (GNU Image Manipulation Program (GIMP) version 2.10.8), because the original area measured may have been inconsistent between eyes. Adjusted 2.3 × 2.3-mm OCTA images in SCP and DCP were binarized according to Niblack’s method with ImageJ (Fig. 1c, f). The flow area in each resulting image was defined as the vascular area, and the number of pixels was quantified with ImageJ software. The VD (%) was calculated using the following formula: VD (%) = vascular area (pixels) / (region of interest – FAZ area) (pixels) × 100.

Foveal minimum thickness (FMT) (μm) was measured by built-in automatic analysis software on the RS-3000 Advance.

### Statistical analyses

All statistical analyses were performed using Bell Curve for Excel version 2.0 software (Social Survey Research Information, Tokyo, Japan). Data are presented as means ± standard deviations or median (interquartile range). For statistical analysis, the decimal BCVA was converted to the logarithm of the minimum angle of resolution (logMAR). Variable normality was inspected using the Shapiro-Wilk test for all variables. The paired t-test or Wilcoxon signed-rank test was used to compare each parameter between the amblyopic eye and the fellow eye. Intraclass correlation coefficient (ICC) was used for the assessment of the inter-rater reliability of FAZ measurements. For all these analyses, values of *p* < 0.05 were considered statistically significant.

## Results

### Demographic data

This study enrolled 15 Japanese patients with anisometropic amblyopia (two patients had anisometropia combined with manifest esotropia). Table 1 shows the demographic and clinical data of patients. The mean age of patients was 9.8 ± 3.4 years. At the time of measuring OCTA, logMAR in amblyopic eyes was significantly poorer as compared to the fellow eye (*p* < 0.001). Refraction was more hyperopic in the amblyopic eye than in the fellow eye (*p* < 0.001). AL was significantly shorter in the amblyopic eye than in the fellow eye (*p* < 0.001).

**Table 1:** Demographic and clinical data of patients at OCTA measurements

	Amblyopic eyes	Fellow eyes	*p*-value
Age			
Mean ± SD	9.8 ± 3.4		NA
Median (range)	9.0 (7.0 to 12.0)		
Sex (Male: Female)	4: 11		NA
Visual acuity (logMAR)			
Mean ± SD	0.21 ± 0.24	−0.17 ± 0.03	< 0.001^a^
Median (range)	0.15 (− 0.02 to 0.40)	−0.18 (− 0.18 to − 0.18)	
Refraction (diopters)			
Mean ± SD	5.13 ± 2.54	1.05 ± 1.96	< 0.001^a^
Median (range)	6.00 (4.25 to 6.88)	1.00 (0.00 to 2.00)	
Axial length (mm)			
Mean ± SD	21.30 ± 1.10	22.78 ± 1.36	< 0.001^b^
Median (range)	21.11 (20.76 to 21.59)	23.14 (21.56 to 23.36)	

N/A: not applicable

Values are shown as mean ± standard deviation and median (interquartile range)

^a^ Wilcoxon signed-rank test; ^b^ paired t-test

### FAZ, VD, and FMT

Inter-observer reliability was excellent for FAZ area measurements. ICCs are expressed with the 95% confidence intervals in parentheses. In the amblyopic eye, ICC was 0.993 (0.924 to 0.998) for SCP and 0.965 (0.902 to 0.988) for DCP. In the fellow eyes, ICC was 0.963 (0.879 to 0.987) for SCP and 0.865 (0.611 to 0.954) for DCP. All ICCs were significantly different from zero (*p* < 0.001).

Table 2 shows the FAZ area, VD, and FMT comparisons between amblyopic and fellow eyes. FAZ area of SCP in amblyopic eyes was significantly smaller than that of fellow eyes (*p* < 0.001). No significant differences were seen in FAZ area of DCP, VD of SCP, VD of DCP, and FMT between amblyopic and fellow eyes (*p* = 0.07, 0.43, 0.55, and 0.25, respectively).

**Table 2:** FAZ, VD, and FMT comparisons between amblyopic and fellow eyes

	Amblyopic eyes	Fellow eyes	*p*-value
FAZ (mm^2^)			
SCP			
Mean ± SD	0.25 ± 0.07	0.30 ± 0.08	< 0.001^b^
Median (range)	0.25 (0.23 to 0.29)	0.32 (0.28 to 0.33)	
DCP			
Mean ± SD	0.31 ± 0.09	0.35 ± 0.09	0.07^b^
Median (range)	0.30 (0.26 to 0.34)	0.33 (0.31 to 0.38)	
VD (%)			
SCP			
Mean ± SD	35.73 ± 0.57	35.88 ± 0.67	0.43^b^
Median (range)	35.68 (35.51 to 35.99)	36.04 (35.56 to 36.36)	
DCP			
Mean ± SD	39.60 ± 0.50	39.68 ± 0.50	0.55^b^
Median (range)	39.74 (39.38 to 39.83)	39.66 (39.32 to 40.04)	
FMT (μm)			
Mean ± SD	227.9 ± 20.5	221.2 ± 10.3	0.25^a^
Median (range)	221.4 (213.1 to 236.1)	221.4 (217.3 to 229.8)	

DCP: deep capillary plexus, FAZ: foveal avascular zone, FMT: foveal minimum thickness, SCP: superficial capillary plexus, VD: vessel density

Values are shown as mean ± standard deviation and median (interquartile range)

^a^ Wilcoxon signed-rank test; ^b^ paired t-test

## Discussion

In the present study, we found that FAZ area of SCP in amblyopic eyes was significantly smaller than that of fellow eyes (*p* < 0.001). There was a trend that FAZ area of DCP in amblyopic eyes was smaller than that of fellow eyes, but the difference was not statistically significant (*p* = 0.07). There was no significant difference in VD or FMT between amblyopic and fellow eyes (*p* > 0.05 for both comparisons).

There have been several reports for OCTA in anisometropic and/or strabismic amblyopia [15–18]. Findings in previous studies are summarized in Table 3. Regarding the FAZ area, most reports have found no significant difference among amblyopic, fellow, or normal control eyes [15, 16, 18]. In contrast, our results showed that FAZ area was smaller in amblyopic eyes compared with fellow eyes. The difference in the results between previous studies and our own could be explained by the presence or absence of magnification correction. Sampson et al. [19] demonstrated the impact of image magnification due to AL variation on VD and FAZ area measurement in OCTA. They reported that the relative change in FAZ area ranged from − 20 to + 51 (% of uncorrected value) after image size correction in healthy subjects with AL of 21.27 to 28.85 mm. Accordingly, image magnification correction for eyes with short AL can make the value of FAZ area smaller. In some previous reports [15, 17, 18] investigating patients with anisometropic amblyopia, the size of FAZ area may have been overestimated due to short AL of the amblyopic eye, as these studies do not appear to have performed magnification correction of the data.

**Table 3:** Previous studies on optical coherence tomography angiography in amblyopia

						FAZ area		VD (3 × 3 mm scan)		
Study	OCTA (algorithm)	Patients with amblyopia	Age (years)	LogMAR of AE	Type of amblyopia	SCP	DCP	SCP	DCP	FMT
Lonngi M, 2017 [15]	RTVue XR Avanti (SSADA)	13	8.0 ± 4.0	0.51 ± 0.17	Anisometropia, strabismus	No difference	No difference	No difference^†^	No difference^†^	No difference
Yilmaz I, 2017 [16]	RTVue XR Avanti (SSADA)	15	8.2 ± 2.3	0.25 ± 0.06	Strabismus	No difference	No difference	Decreased in AE^‡^	Decreased in AE^‡^	–
Sobral I, 2018 [17]	RTVue XR Avanti (SSADA)	26	9.2 ± 2.8	0.18 ± 0.21	Anisometropia, strabismus	No difference	Increased in AE^‡^	Decreased in AE	No difference	No difference
Demirayak B, 2019 [18]	RTVue XR Avanti (SSADA)	17	8.6 ± 2.5	0.32 ± 0.06	Anisometropia, strabismus	No difference	–	No difference	No difference	No difference
Our study	NIDEK RS-3000 Advance (CODAA)	15	9.8 ± 3.4	0.21 ± 0.24	Anisometropia, mixed	Decreased in AE	No difference	No difference^§^	No difference^§^	No difference

AE: amblyopic eyes, CODAA: complex OCT-signal difference analysis angiography, DCP: deep capillary plexus, FAZ: foveal avascular zone, FMT: foveal minimum thickness, LogMAR: logarithm of the minimum angle of resolution, OCTA: optical coherence tomography angiography, SCP: superficial capillary plexus, SSADA: split-spectrum amplitude-decorrelation algorithm, VD: vessel density

^†^ VD in a 6 × 6-mm area is decreased in amblyopic eyes. ^‡^ No significant difference is evident between amblyopic and fellow eyes, but a significant difference is present between amblyopic and normal control eyes. § The analysis region of the OCTA image was 2.3 × 2.3-mm after magnification correction. Values are shown as mean ± standard deviation

On the other hand, there are reports that FAZ area was small or absent in premature infants and patients with foveal hypoplasia [22, 23]. The formation of FAZ is thought to be critical in the development of the foveal pit. If FAZ fails to form, the inner retinal layers may persist in the foveal center, and the foveal pit becomes shallow [24]. However, despite the difference in FAZ between amblyopic and fellow eyes in this study, the FMT of the amblyopic eye was not different from that of fellow eyes. Therefore, the mechanism for the small FAZ area in amblyopic eye found in present study appears different from that of hypoplasia of FAZ seen in premature infants and patients with foveal hypoplasia.

Our present study did not include healthy control eyes and it remains unclear whether fellow eyes of patient with unilateral amblyopia can be regarded as completely normal. However, previous studies [16–18, 21] have shown that FAZ areas of healthy children are 0.19 to 0.33 mm^2^ in the SCP and 0.22 to 0.33 mm^2^ in the DCP. Although comparing measured values derived from different OCTA models is difficult [25], the FAZ area in the amblyopic or fellow eye in our study did not appear to deviate from that of healthy eyes. Therefore, the small FAZ found in the amblyopic eye in this study is unlikely to be clinically significant.

Regarding VD, some reports have found decreased VD in amblyopic eyes [15–17], while another found no change in amblyopic eyes [18]. Our present study supports the latter results [18] that the VD of amblyopic eyes is not altered. In normal healthy children, Cheung et al. [26] found that a decreased AL was associated with decreased VD. They considered the finding most likely due to the magnification error. Our study corrected for magnification errors in OCTA image using individual AL. This could have resulted in a difference in the results between previous studies [15–17] and our own. In addition, difference in VD regions of interest across studies may also affect differences in the results.

Several limitations associated with the present study need to be considered. First, we could not remove projection artifacts at the level of the DCP. Thus, the possibility remains that vessels distributed in the SCP may have been included in the analysis of the DCP structure, and care should be taken in interpretation of the results for VD in the DCP. Second, only a small number of patients was included in this study. Furthermore, as most cases of amblyopia in this study were caused by anisometropia, our findings may not apply to purely strabismic amblyopia or deprivation amblyopia. In addition, most patients studied here were mild amblyopes. Whether the current results hold true for severe amblyopia is thus unclear. Further study including a larger number of amblyopic patients is necessary to support the results of our study.

## Conclusion

In conclusion, we found smaller FAZ area of SCP in amblyopic eye compared with that of fellow eyes, but there was no significant difference in macular VD between amblyopic and fellow eyes, when the data were corrected for magnification error.

## Data Availability

The datasets used and/or analysed during the current study are available from the corresponding author on reasonable request.

## Abbreviations

AL: Axial length
BCVA: Best corrected visual acuity
CI: Confidence interval
DCP: Deep capillary plexus
DR: Diabetic retinopathy
FAZ: Foveal avascular zone
FMT: Foveal minimum thickness
ICC: Intraclass correlation coefficient
logMAR: Logarithm of the minimum angle of resolution
OCT: Optical coherence tomography
OCTA: Optical coherence tomography angiography
RVO: Retinal vein occlusion
SCP: Superficial capillary plexus
SD-OCT: Spectral-domain optical coherence tomography
VD: Vessel density
